# Establishing Safe Working Parameters for Radiofrequency Ablation In Vitro Using Acoustic Sensing, Probability Mapping, and Catheter Contact Angle

**DOI:** 10.19102/icrm.2022.130703

**Published:** 2022-07-15

**Authors:** Wadih El Khoury, Joseph Al Aaraj, Anthony Gebran, Marwan Hajjar, Rawad Abbas, Hussein Daoud, Maurice Khoury, Bernard Abi-Saleh, Ghanem F. Oweis, Marwan M. Refaat

**Affiliations:** ^1^Department of Mechanical Engineering, Faculty of Engineering, American University of Beirut, Beirut, Lebanon; ^2^Faculty of Medicine, American University of Beirut, Beirut, Lebanon; ^3^Department of Internal Medicine/Cardiology Division, Faculty of Medicine, American University of Beirut, Beirut, Lebanon

**Keywords:** Acoustic sensing, cardiac arrhythmias, cardiovascular diseases, catheter ablation, heart diseases, steam pop

## Abstract

Surgical quality and safety in radiofrequency catheter ablation (RFA) are critical in arrhythmia procedures. Steam pops, in particular, are potentially catastrophic events that must be avoided; otherwise, they may cause significant damage to the myocardium. This study aimed to evaluate the effect of applied RFA inclination angle and tissue contact parameters on the ablated volume and “steam pop” formation. An ex vivo model consisting of a viable ovine myocardium, an ablation catheter, and a circulating warmed 0.9% NaCl saline solution was used. RFA was conducted while controlling for contact force, electrical power, ablation time, flow rate, irrigation, and catheter tip angle. Irrigation was delivered to the catheter tip manually when indicated. Acoustic transducers were included in the setup to detect preliminary acoustic signals. A total dataset of 567 measurements was taken. Benign precursory signals (hissing and lower-intensity “pops”) were detected by acoustic sensors preceding the occurrence of “steam pops.” Furthermore, a Pearson coefficient of *r* = 0.809 with *P* < .01 was shown to exist between the acoustic intensity of a “steam pop” and the ablated lesion volume. RFA powers of 25 and 30 W with a duration of 20 s induced more “steam pops” than ablation powers of ≤20 W with a duration of ≥30 s. There was also an increased probability of “steam pop” formation with the use of a non-irrigated catheter tip, as compared to an irrigated catheter tip. A more acute catheter angle increased the lesion size at powers of 20 and 25 W (*r* = −0.568 and *r* = −0.653, both *P* < .05, respectively). There is a potential benefit of using acoustic sensing as a warning before the occurrence of “steam pops.” Varying power, duration, and catheter tip angle will generate different ablation sizes and need to be tailored to individual needs and procedures.

## Introduction

Cardiac arrhythmias are associated with increased morbidity and mortality worldwide.^1–3^ Management may incorporate anti-arrhythmic medications, which are sometimes effective but carry the risk of heavy side effects.^4,5^ Non-pharmacological management includes radiofrequency (RF) catheter ablation (RFA) and cryoablation.^6–9^

During RFA, the catheter is introduced through the venous or arterial system via the femoral vessels or the internal jugular and subclavian veins. The catheter tip is maneuvered to the ectopic focus or any other site of interest.^10–12^ A medium-frequency alternating current in the range of 300–1,000 kHz is delivered by the catheter tip to ablate the abnormal tissue.^13^ Irrigation of the catheter tip with normal or half-normal saline is implemented for cooling, providing longer ablation time, and enabling the formation of larger lesions.^14–16^ Irrigation also delays the formation of “steam pops” and possibly prevents their formation.^17–19^ “Steam pops” arise due to fast and localized tissue heating (>100°C) and result in adverse outcomes, mainly potential thromboembolic complications and myocardial perforation leading to cardiac tamponade.^17,20–23^ The ablation process and “steam pop” formation depend on multiple variables, including the size of the catheter tip, electrical power, contact force, angular orientation, and time of application.^24–27^

Few studies looked at the catheter angle of application (other than 0° and 90°)^28–30^ in determining its effect on “steam pop” formation. Furthermore, the use of acoustics is a promising emerging field to detect precursory warning signals (hissing and precursory low-intensity “pops”) that prevent “steam pops” and warrants further research and investigation.^31,32^ The aim of this study was to understand how power, irrigation, myocardial tissue thickness, and catheter angle affect the ablation dynamics and “steam pop” formation and to study precursory acoustic signals and their potential benefit in avoiding detrimental lesions to the myocardium.

## Methods and experimental setup

### Ablation parameters and conditions

In this study, the ovine myocardium was divided into left and right ventricular tissues. The dissected ovine heart tissue, butchered 8–12 h earlier and refrigerated at 5°C–7°C, was treated with RFA ex vivo to study the formation of ablation lesions and “steam pop” parameters. A custom-designed setup was built to investigate the ablation process while controlling for contact force between the catheter and the ovine specimen (kept constant at 20 g), electrical power (15, 20, 25, 30, and 35 W), ablation time (20, 30, 40, 50, 60, and 70 s), flow rate of the 0.9% NaCl saline solution coming out from the tip of the RF catheter, and catheter tip angle (measured between the catheter tip and the ovine specimen placed horizontally). The specimen was submerged in a flowing saline bath emulating ventricular blood flow. The whole bath system floated on load cells to quantify the applied catheter contact force.

### Experimental setup

The experimental setup consisted of an ablation catheter (ThermoCool SF Irrigated Catheter; Biosense Webster, Diamond Bar, CA, USA) having a cylindrical tip with a diameter of 3.5 mm. The tip of the catheter had multiple small openings on its sides allowing for the passage of the irrigation fluid (saline solution, 0.9% NaCl concentration). The irrigation flow was manually driven using a syringe with a nearly constant flow rate of 0.5 mL/s. The catheter was attached to a tilting mechanism to control the angle between the catheter tip and the specimen. In all measurements, the catheter was positioned perpendicular to the myocardial tissue unless indicated otherwise. RF power to the catheter was provided via an RF generator (OSYPKA HAT 200 S: Osypka Occluder GmbH, Grenzach-Wyhlen, Germany).

The ventricular blood-flow surrogate (saline, 0.9% NaCl) was driven by a centrifugal pump (5 W, RS-2002; R S Electrical, Northamptonshire, UK) at a flow rate of 50 mL/s. The flow temperature was kept close to 37°C using a feedback-control heater (OMEGA MAX™; Racold Wateer Heater, Chennai, India) with a Type K thermometer (BK PRECISION 715; B & K Precision, Yorba Linda, CA, USA).

**Figure 1:**
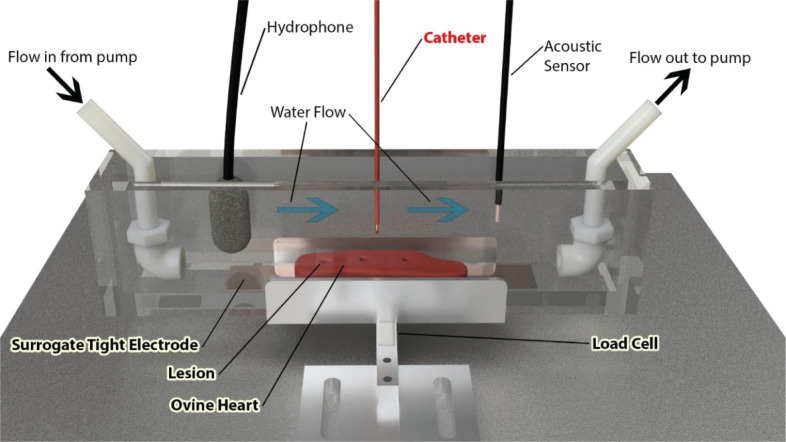
Experimental setup rendering showing the plexiglass container placed on a load cell to measure the force applied. The ovine myocardium is placed in the center of the container while being constantly irrigated by the saline solution at 37°C. The catheter is directly positioned above the myocardium and is labeled in red. On the sides of the container, an acoustic sensor and a hydrophone were installed in order to sample the acoustic data.

The contact force was measured using a load cell set accompanied with an amplifier (SMOWS RW-ST01A; SMOWS, Shanghai, China). A calibration procedure produced a calibration curve to convert the readout voltage to contact force **(Figure 1)**.

### Acoustic signals measurement setup

Acoustic emissions were monitored using a high-frequency hydrophone (SONIC CONCEPTS Y-104/D, 1.9 MHz; Sonic Concepts Inc., Bothell, WA, USA) and a low-frequency acoustic transducer (<60 kHz) **(Figure 1)**. The low-frequency transducer consisted of a commercial computer microphone that was custom water-proofed using potting room-temperature-vulcanizing silicone. Two different sampling frequencies were used for the 2 devices so that the low-frequency transducer produced a panoramic recording of the entire acoustic event (50 seconds), and the high-frequency hydrophone was auto-triggered by the primary steam pop event (35 ms = 5 ms pre-trigger + 30 ms post-trigger). The 2 signals were monitored on the same time base.

### Ablation lesion size measurement

**Figure 2:**
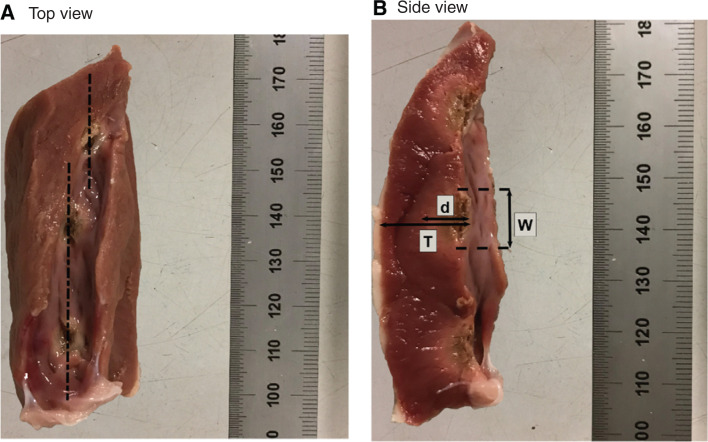
Two views of the left ventricular tissue after radiofrequency ablation at 3 distinct sites at a power of 35 W, a force of 20 g, and an exposure time of 50 s for each site using an irrigated catheter at 90° above the tissue (perpendicular to it). **A:** The tissue is shown from a top view after ablation. **B:** The lesion depths after midline incision through the lesions with 90° clockwise rotation are shown. *W* is the width of the ablation lesion on the surface, *d* is the depth of the ablation lesion, and *T* is the total thickness of the specimen at the location of ablation.

By approximating the burn geometry to a conical shape and measuring the radius 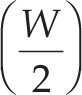 and depth (*d*) **(Figure 2)** of the ablation lesion using the images enhanced on MATLAB (MathWorks, Natick, MA, USA), the volume of the lesions (*V*) was computed using the following formula: 
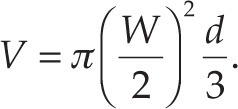
 In order to eliminate the effect of varying thicknesses along the different myocardial tissues, a normalized set of data needs to be created. For this reason, during ablation, the ratio between the depth of the ablation lesion (*d*), measured vertically from the point of contact of the catheter tip to the deepest point of the ablated lesion (moving from the epicardium to the myocardium vertically) was measured and then divided by the local thickness of the ventricular tissue (*T*) **(Figures 2A and 2B)**. The ratio expression is therefore 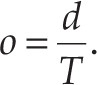 This measurement allowed the depth of each lesion to be standardized to the total depth of the myocardium where it occurred. Hence, a lesion ratio of 0.6 would mean that the ablated site was able to penetrate 60% of the total thickness of the ventricle in that particular specimen. The same process was carried out for the right ventricular tissue.

### Statistical analysis

Statistical analysis was conducted using SPSS Statistics version 23 (IBM Corporation, Armonk, NY, USA). The ablation ratio and occurrence of “steam pop” were correlated to the power and exposure time of the RFA using Pearson’s correlation coefficient with *P* < .05 (or, in some cases, *P* < .01) when specified. It is important to note that, when the power is used to derive a correlation between it and the ablation ratio or “steam pop” occurrence, time is kept constant and only power is varied. Hence, only a single variable is altered at a time. The opposite is true; when time is varied to understand its effect on “steam pop” formation or ablation ratio, the power of the RFA catheter is kept constant.

The contour probability plots, discussed in detail later, were defined and calculated as follows: for every combination of power and time, 5 ablation lesions were made, each with the same power and time of ablation. For each ablation lesion, the occurrence of “steam pop” was noted. Then, the number of total “steam pop” occurrences was divided by 5, which is the total number of lesions taken for those specific power and time couple. Hence, for a certain combination of power and time, a percentage of “steam pop” occurrence could be determined with its percentage varying between 0%–100%. For combinations of power and time where no measurements were made, an interpolation between known points was used. This allowed the team to develop a topological map with the x-axis and y-axis representing the power and time parameters, respectively, and the z-axis denoting the probability of a steam pop occurring for a specific combination.

## Results

### Acoustic detection

**Figure 3:**
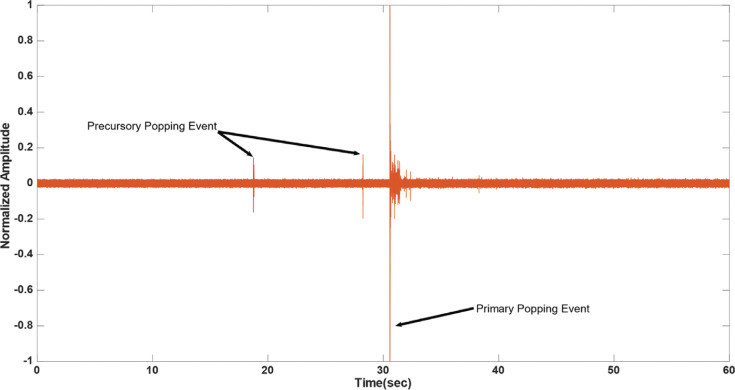
Plot of the normalized acoustic signal sampled from the hydrophone for left ventricular radiofrequency catheter ablation using an irrigated catheter placed at 90° at a power of 15 W, an exposure time of 60 s, and a force of 20 g. Two lower-amplitude precursory signal “steam pops” occurred at 10 and 3 s, respectively, before the damaging “steam pop” represented by a higher amplitude. These could be used as a warning signal.

**Figure 4:**
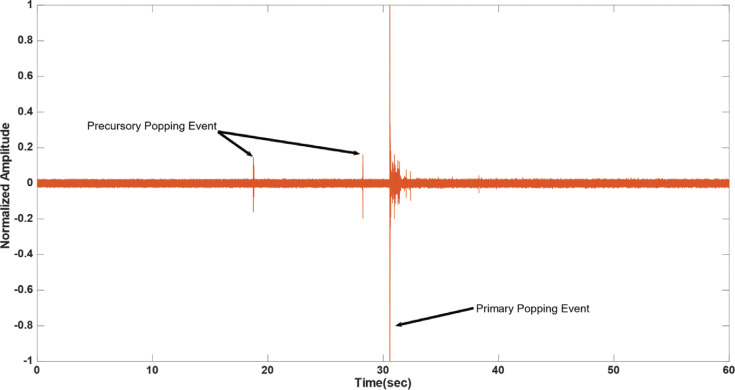
Plot of the normalized acoustic signal sampled from the hydrophone for left ventricular radiofrequency catheter ablation using an irrigated catheter placed at 90° at a power of 15 W, an exposure time of 60 s, and a force of 20 g. A lower-amplitude precursory signal “steam pop” and “hissing,” due to pressure release, were noted 4 s before the onset of the damaging “steam pop.”

In several of the observed cases, lower-intensity “micro-pops” preceded the irreversible damage incurred to the myocardium by a “primary steam pop.” These “micro-pops” had a substantially (80%) lower amplitude compared to the damaging “steam pop.” These occurred 3–10 s before the “steam pop” **(Figure 3)** and could be distinguished by the human ear, in addition to the low-frequency acoustic transducer. In the minority of cases (20.7%), audible “hissing” could be identified, which suggested micro-events of steam buildup and release. Hissing lasted between 5–15 s **(Figure 4)**. Precursory “pops” were observed in 27.6% of the cases, and these could be described as localized acoustic events that are benign in comparison to the primary pop, but with a peak that rises above the background hissing level. In 44.8% of the cases, it was possible to detect a precursory signal to the primary steam pop, i.e., a “hissing” sound, a precursory “pop,” or both, with a positive predictive value of 0.833.

**Figure 5:**
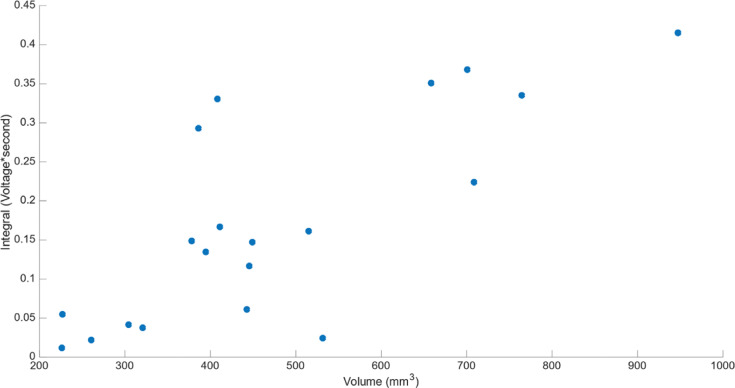
Plot of the integral of the absolute values of the acoustic signal from the microphone for any “steam pop” (the absolute function was implemented in order for the negative values not to cancel the positive one and then integrated to get the intensity of the steam pop) on the y-axis, represented by a blue circle, against the area of the ablated lesion multiplied by the lesion depth, thus volume, on the x-axis. In total, 20 ablation lesions were made to plot this figure.

The intensity of the acoustic signal emanating from the “steam pop” was quantified by the time integral of the acoustic signal (absolute value). A higher-intensity “steam pop” significantly correlated with the lesion volume **(Figure 5)** with a Pearson coefficient of *r* = 0.809 and *P* < .01.

### Ablation dynamics and contour probability

**Figure 6:**
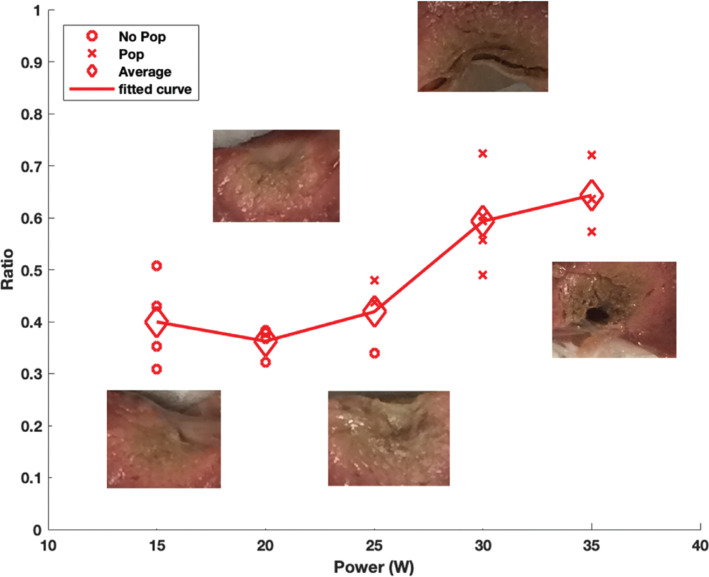
Plot of the ratio of the ablated lesion versus power with a representative scaled lesion for the corresponding ablation site. Done for a radiofrequency catheter ablation with an irrigated catheter placed at 90° above the myocardial tissue with a constant force of 20 g and an exposure time of 50 s. The “o” represents lesions that did not incur a “steam pop,” and the “×” represents lesions that did. The diamond-shaped icons are the average of the ratio for the points sharing the same power. In total, 19 ablation lesions were made. A line was then plotted connecting these averages to get a better understanding of the overall trend.

The lesion ratio was directly proportional to power (0.4 at 15 W; 0.65 at 35 W) with a Pearson correlation factor of *r* = 0.825 and *P* < .01 **(Figure 6)**. Furthermore, increasing power resulted in increasing “steam pop” formation with *r* = 0.871 and *P* < .01.

A prolongation in ablation time resulted in the increased probability of “steam pops” for irrigated left ventricular tissue. Taking data points having the same power, the ablation time was varied from 20–70 s with increments of 10 s. For each power value chosen, a correlation coefficient was calculated, which described the relationship between increasing time and “steam pop” formation at a constant power. Then, this same procedure was repeated for every other power level (15, 20, 25, 30, and 35 W). The results are presented in **Table 1**, showing the correlation coefficient along with its corresponding significance level. All results show a positive correlation between “steam pop” formation and an increase in the time of ablation at all power levels.

In what follows, *T* will be used to indicate the time of ablation, or the time the catheter tip was in contact with the tissue while heating was active. *P* will refer to the power setting of the catheter during ablation. In addition, the probability of “steam pops” is defined as the fraction of occurring “steam pops” over the total number of trials done given a specific setting configuration in an experiment (eg, given power, time).

**Figure 7:**
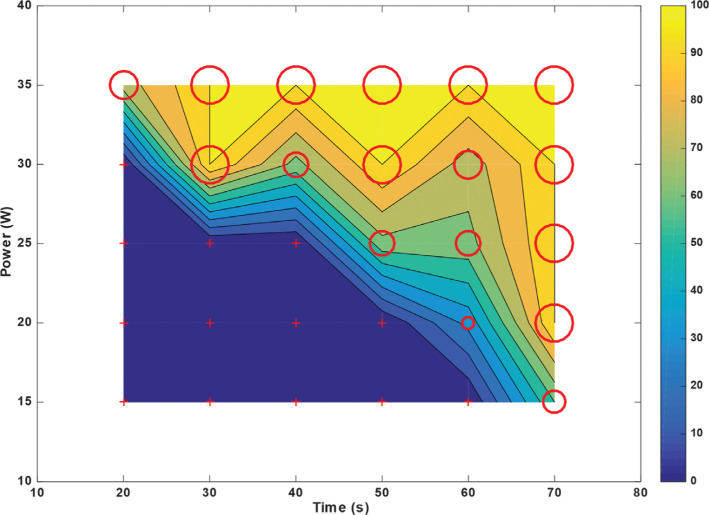
Probability of “steam pop” formation for left ventricular radiofrequency catheter ablation with an irrigated catheter placed at 90° above the tissue for a constant force of 20 g with varying exposure times on the x-axis and power levels on the y-axis. Each point on the plot marked by a “+” or by a circle of variable diameter represents the average of 5 ablations, and, from these, a probability was calculated; in total, this figure represents 150 ablation lesions (5 for each power and time coordinate, with 30 different combinations). The larger the diameter, the higher the probability of “steam pop” formation. The “+” indicates zero probability. The contour ranging from blue (minimum) to yellow (maximum) shows the probability between the sampled points.

**Figure 8:**
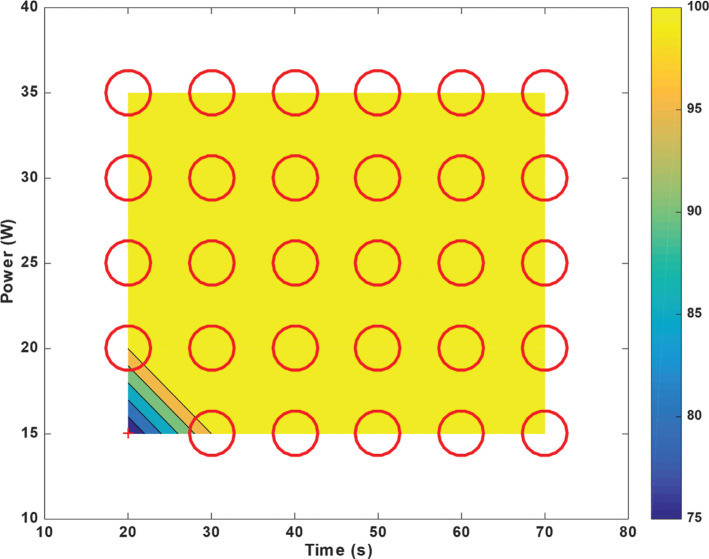
Probability of “steam pop” formation for left ventricular radiofrequency catheter ablation using a non-irrigated catheter placed at 90° above the tissue for a constant force of 20 g with varying exposure times on the x-axis and power levels on the y-axis. Each point on the plot marked by a “+” or by a circle of varying diameter represents the average of 5 ablations, and, from these, a probability was calculated; in total, this figure represents 150 ablation lesions (5 for each power and time coordinate, with 30 different combinations). The larger the diameter, the higher the probability of “steam pop” formation. The “+” indicates zero probability. The contour ranging from blue (minimum) to yellow (maximum) shows the probability between the sampled points.

For *T*_max_ = 70 s at *P*_min_ = 15 W, the probability of “steam pops” hovered at 50%. However, for *T*_min_ = 20 s at *P*_max_ = 35 W, this probability increased to ≈70% **(Figure 7)**. In contrast, for non-irrigated left ventricular tissue, “steam pops” occurred for any power > 20 W and any time > 30 s; a documented macroscopic change in the myocardial tissue was recorded **(Figure 8)**.

**Figure 9:**
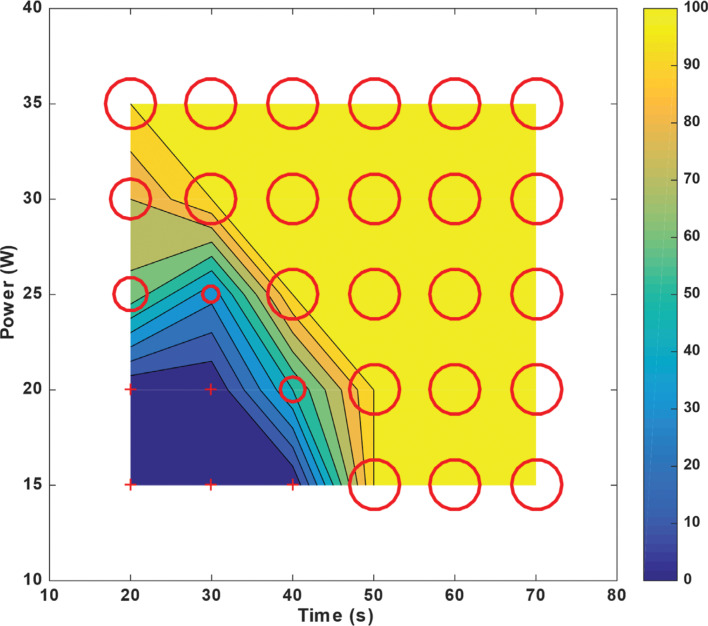
Probability of “steam pop” formation for right ventricular radiofrequency catheter ablation with an irrigated catheter placed at 90° above the tissue for a constant force of 20 g with varying exposure times on the x-axis and power levels on the y-axis. Each point on the plot marked by a “+” or by a circle of varying diameter represents the average of 5 ablations, and, from these, a probability was calculated; in total, this figure represents 150 ablation lesions (5 for each power and time coordinate, with 30 different combinations). The larger the diameter, the higher the probability of “steam pop” formation. The “+” indicates zero probability. The contour ranging from blue (minimum) to yellow (maximum) shows the probability between the sampled points.

For irrigated right ventricular tissue, a similar trend was observed in comparison to the left ventricle, albeit with lower thresholds. For *T*_max_ = 70 s at *P*_min_ = 15 W, the probability of “steam pops” was 100%; for *T*_min_ = 20 s at *P*_max_ = 35 W, this probability was ≈95%. Also, for any ablation time ≥40 s, “steam pops” were recorded regardless of power **(Figure 9)**. Similarly, for non-irrigated right ventricular tissue, “steam pops” were inevitable even with the lowest parameters.

### Angle of the catheter

**Figure 10:**
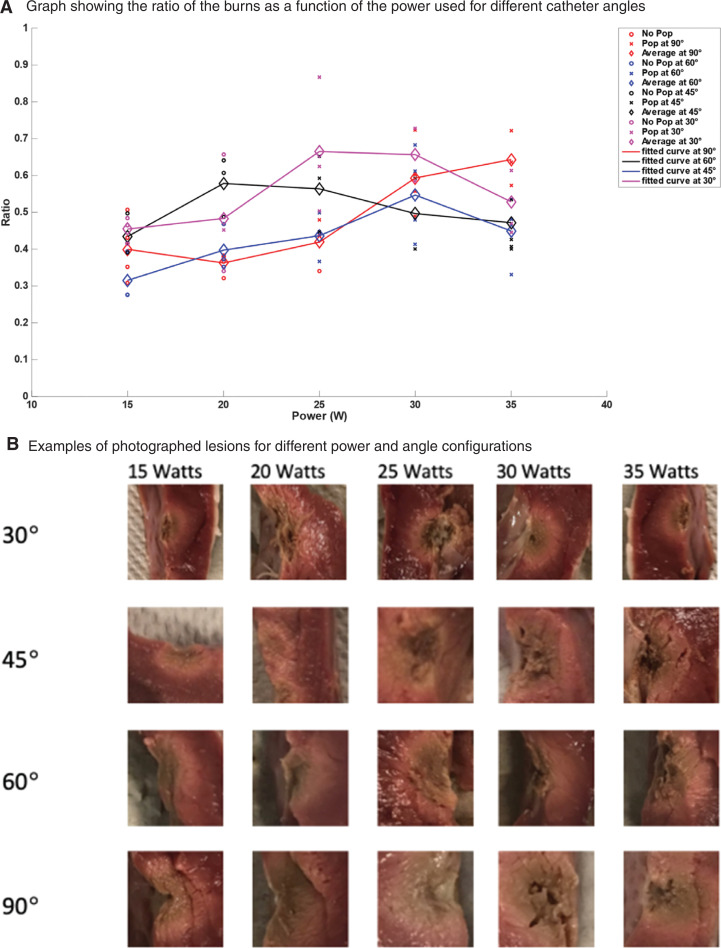
**A:** Plot of the ratio of the ablated lesion versus power. This was done for left ventricular radiofrequency catheter ablation using an irrigated catheter placed at 90°, 60°, 45°, and 30°, taking the horizontal line as the initial starting position, for a constant force of 20 g and an exposure time of 50 s. The “o” represents lesions that did not incur a “steam pop,” and the “×” represents the ones that did. The diamond-shaped icons are the average of the ratio for the points sharing the same power. A line was then plotted connecting these averages in order to get a better understanding of the overall trend. In total, 60 ablation lesions were made to plot this figure. **B:** The representative scaled ablation sites for angles ranging from 30°–90° at powers ranging from 15–35 W are displayed.

**Figure S1:**
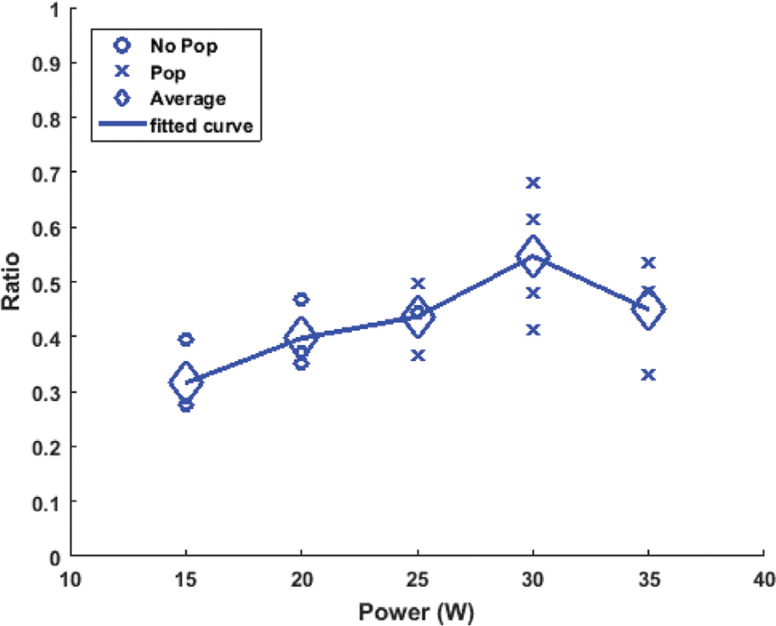
Plot of the ratio of the ablated lesions versus power. This was done for left ventricular radiofrequency ablation with an irrigated catheter placed at 60° above the tissue, taking the horizontal line as the initial starting position, with a constant force of 20 g and exposure time of 50 s for each ablation site. The “o” represents lesions that did not incur a “steam pop” and the “x” represents the ones that did. The diamond-shaped icons are the average of the ratio for the points sharing the same power. In total, 15 ablation lesions were made. A line connecting these averages was then plotted in order to get a better understanding of the overall trend.

**Figure S2:**
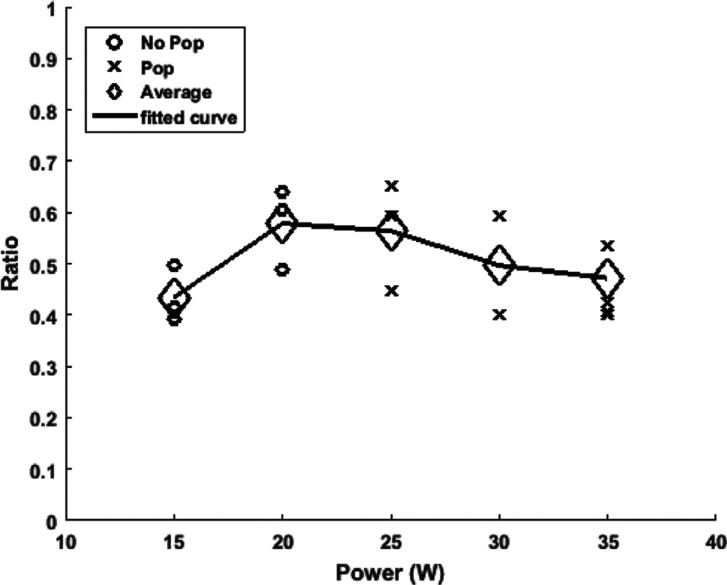
Plot of the ratio of the ablated lesion versus power. This was done for left ventricular radiofrequency ablation with an irrigated catheter placed at 45° above the tissue, taking the horizontal line as the initial starting position, with a constant force of 20 g and exposure time of 50 s for each ablation site. The “o” represents lesions that did not incur a “steam pop” and the “x” represents the ones that did. The diamond-shaped icons are the average of the ratio for the points sharing the same power. In total, 15 ablation lesions were made. A line was then plotted connecting these averages in order to get a better understanding of the overall trend.

**Figure S3:**
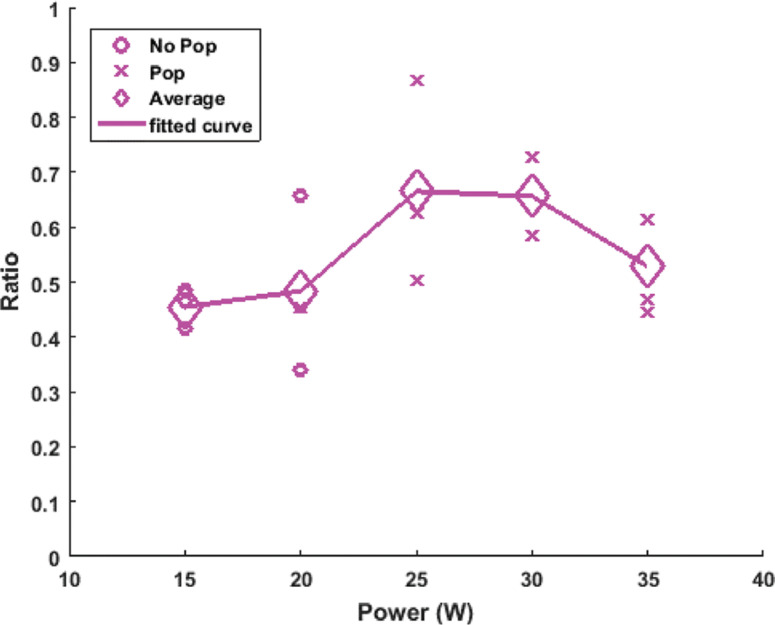
Plot of the ratio of the ablated lesions versus power. This was done for left ventricular radiofrequency ablation with an irrigated catheter placed at 30° above the tissue, taking the horizontal line as the initial starting position, with a constant force of 20 g and exposure time of 50 s for each ablation site. The “o” represents lesions that did not incur a “steam pop” and the “x” represents the ones that did. The diamond-shaped icons are the average of the ratio for the points sharing the same power. In total, 14 ablation lesions were made. A line connecting these averages was then plotted in order to get a better understanding of the overall trend.

For a constant ablation time of 50 s, “steam pops” started to occur at 25 W for all catheter angles except 30°, where “steam pops” were recorded starting at 20 W. As an observation, the occurrence of “steam pops” was minimally affected by the change in the catheter angle, as 10 of 11 data points recorded “steam pops” at 25 W irrespective of the catheter angle **(Figure 10A)**. Taking the power level at 25 W, in order to observe the effect of catheter angle on the ablation ratio, it was seen that at angles of 90°, 60°, 45°, and 30°, the average ablation ratio values were 0.42, 0.44, 0.56, and 0.66, respectively. Thus, a more acute angle produced a higher ablation ratio, as this was especially true for power levels of 20 and 25 W **(Figure 10B)** with the respective Pearson coefficients being *r* = −0.568 and *r* = −0.653, both for *P* < 0.05. Individual angle studies can be found online in **Figures S1–S3 and Table S1**.

## Discussion

### Acoustics

The main aim of this study was to assess the relevance of acoustic sensing to “steam pop” forecasting and, consequently, mitigation. In most of the cases, the hydrophone used in the experimental setup detected precursory signals (“steam pops” and “hissing”) in the order of seconds. The occurrence of those emissions often preceded “steam pop” formation **(Figures 3 and 4)**. Therefore, those precursory signals can serve as a warning sign to the electrophysiologist of an impending “steam pop,” potentially preventing its occurrence.

These findings are in accordance with those of Chik et al.,^31^ who demonstrated, in vitro, the importance of using a hydrophone to detect acoustic warning signals. It was reported that those signals were identified as frequently as 80% of the time before “steam pop” occurrence.^31^

The positive Pearson correlation reported in the Results section and seen between the intensity of the acoustic signal and the ablated lesion volume indicates that, indeed, the stronger the acoustic signal, the bigger the ablated volume. For instance, for intensities of 0.0114 and 0.4149 V, the volumes of the ablation lesion were 226.3 and 947.3 mm^3^, respectively.

### Ablation dynamics and contour probability

Any study of the RF ablation parameters should include a safety assessment. Our main concern here is to minimize the formation of “steam pops” and subsequent myocardial damage. For a constant energy (power × time) of 600 J, ablating the right ventricular tissue at 20 W for 30 s resulted in 0% probability of “steam pops.” In contrast, ablating at 30 W for 20 s resulted in ≈75% probability of “steam pops” **(Figure 7)**. A similar trend was observed in the left ventricular tissue for a constant energy. Hence, an RFA power of ≥25 W with ablation times <20 s induced more “steam pops” than an ablation power of ≤20 W with durations of ≥30 s. A recent publication by Borne et al. evaluated ablation lesion size with increasing power delivery or longer ablation time. Using both ex vivo and in vivo models, they demonstrated that, with a constant force, ablation size increased in a proportional way to power, but only half as much for RFA duration.^33^ In other words, ablation power had a stronger influence than ablation time on lesion size. While “steam pop” probability chart values might not be directly applicable in clinical settings, similar trends might be observed in vivo. This study was neither designed nor powered to evaluate clinical endpoints; therefore, these findings should be further evaluated in randomized trials. To note, the number of “steam pops” recorded in this study was higher than that detected in a clinical setting with similar parameters.^34^ This could be explained by the limitations mentioned below.

When it comes to the probability of “steam pop” formation, left and right ventricular tissues behaved differently. The right ventricle is thinner and less muscular than the left ventricle, which means that the right ventricle has a lower impedance.^35^ When a tissue with a lower impedance is charged with a fixed power, a larger current will be generated, leading to a higher probability of tissue damage. Thus, it is expected that “steam pop” formation would occur at a lower RFA power and duration in the right myocardium compared to the left **(Figures 7 and 9)**. Irrigated left ventricular tissue was particularly resistant to injury and the probability of “steam pop” formation remained at <50% for an RFA power of <30 W **(Figure 7)**.

Myocardial tissue was affected differently when subjected to irrigation. Irrigation of the catheter’s tip delayed or even prevented “steam pop” formation by cooling the tissue, rendering it a protective factor, in accordance with the report of Müssigbrodt et al.^36^ A prospective randomized trial by Pérez-Castellano et al. compared the efficacy of irrigated catheter ablation to standard catheter ablation in achieving pulmonary vein conduction block in patients with atrial fibrillation. Irrigated ablation reduced the operative time and resulted in less frequent recurrence of AF as well as decreased the rates of pulmonary vein stenosis.^37^ Nguyen et al. assessed the efficacy and safety of half-normal saline in the treatment of ventricular arrhythmias refractory to standard ablation with normal saline irrigation. RF delivery using half-normal saline was effective and successful in the ablation of deep myocardial tissue substrates.^19^

### Angle of the catheter

In this study, smaller angles (30°, 45°) resulted in a greater lesion ratio compared to larger angles (60°, 90°), with up to 1.5 times larger lesions with small angles at low power levels (20 and 25 W). This finding could be explained by the higher contact surface that results between the catheter tip and the myocardial tissue at more acute angles, and, hence, this resulted in a larger lesion as predicted. Although an in vivo model was not used, the determination of angle effect is more challenging in this model, as application of exact angles might be harder to achieve and warrants further research. These results were corroborated by the findings of Gallagher et al.^28,30^ Using contact angles ranging from 15°–90°, it was found that more acute catheter angles caused larger lesions with up to a 2-fold difference in size between the angle extremes.^28–30^

### Study limitations

In this paper, the authors recognize several limitations to this study. First, ovine myocardium was used for the purposes of the research, which may have different physiological properties compared to human myocardium, and this myocardium was not perfused by blood unlike a functional myocardium, which could provide internal tissue cooling to the myocardium. Second, in these experiments, the ovine tissue used was static and did not simulate the normal beating motion. Third, only ventricular tissue was used, extrapolation to outcomes in atrial tissue is cautioned against pending further investigation. Moreover, “steam pop” probability charts mentioned in this study might not be applicable to in vivo models; therefore, further investigations are needed. This study is done with a constant contact force value in favor of variable catheter angle, power, time, and irrigation status. Unfortunately, the utilized device did not provide for catheter tip temperature sensing.

The acoustic environment in this study is significantly different from that within a patient, and an acoustic detection call of steam popping might not correspond directly to a clinical call of steam pop event. In addition, the whole catheter ablation setup, which consists of an RF catheter, a hydrophone, and an acoustic sensor, cannot be inserted during an actual clinical procedure. This warrants the development of RF catheters that incorporate within them acoustic sensors.

## Conclusion

In this study, there is a special emphasis on the potential preventive role of acoustic sensing during any cardiac RF ablation procedure. The use of a hydrophone allows the potential detection of precursory signals in the order of seconds before “steam pop” formation. Those signals can be used as a novel method of preventing the occurrence of myocardial damage. This study also explores and establishes safe working parameters for electrophysiologists during cardiac catheterization by proposing a probability map delineating safe regions of operation.

## Figures and Tables

**Table 1:** Statistical Analysis Values for the Correlations Seen Between Varying Times and “Steam Pop” Formation for a Given Constant Power Value

Power (W)	Pearson Coefficient *r*	Significance Level *P*
15	0.533	<.05
20	0.715	<.01
25	0.776	<.01
30	0.503	<.05
35	0.462	<.05

The analysis was then repeated for several power levels. Ablation time was varied from 20–70 s in increments of 10 s at each power level. The results presented show a positive correlation between “steam pop” formation and ablation time at all power levels.

**Table S1:** Ratio of the Ablated Lesions Versus Power

Specimen Number	Lesion Number	Power (W)	Thickness of Ventricle *T* (mm)	Lesion Depth *d* (mm)	Width (mm)	Ratio (*d/T*)	Steam Pop Occurrence
1	1	15	11.1464	5.6489	4.2207	0.50679143	no
	2	15	12.4708	3.847	5.6536	0.308480611	no
2	1	15	10.088	4.344	3.68	0.430610626	no
	2	15	12.083	4.255	4.796	0.352147645	no
3	1	20	10.678	3.92	4.146	0.367109946	no
	2	20	12.879	4.14	5.759	0.321453529	no
	3	20	14.464	5.48	5.989	0.378871681	no
	4	20	11.876	4.543	6.123	0.382536207	no
4	1	25	14.9236	5.0719	5.5751	0.339857675	no
	2	25	13.2782	6.3744	2.8813	0.480065069	yes
	3	25	11.2927	4.9382	3.975	0.437291348	yes
5	1	30	16.9844	9.4554	5.5759	0.556710864	yes
6	1	30	10.585	6.38	4.436	0.602739726	yes
	2	30	10.586	5.186	2.817	0.489892311	yes
	3	30	10.238	7.409	3.288	0.723676499	yes
	4	30	11.739	6.958	5.643	0.592725104	yes
7	1	35	13.5812	9.777	4.9709	0.719892204	yes
	2	35	13.5114	11.4647	8.0096	0.848520509	yes
	3	35	12.5487	9.3026	7.7774	0.741319818	yes

Done for a radiofrequency ablation procedure with an irrigated catheter placed at 90° above the myocardial tissue with a constant force of 20 g and exposure time of 50 s. In total, 19 ablation lesions were made. Pearson’s correlation between power and ratio had a coefficient r = 0.825 with a significance level *P* < .01. The correlation between power and steam pop formation had a coefficient r = 0.871 with a significance level *P* < .01.
